# Live Biotherapeutic Products, A Road Map for Safety Assessment

**DOI:** 10.3389/fmed.2020.00237

**Published:** 2020-06-19

**Authors:** Alice Rouanet, Selin Bolca, Audrey Bru, Ingmar Claes, Helene Cvejic, Haymen Girgis, Ashton Harper, Sidonie N. Lavergne, Sophie Mathys, Marco Pane, Bruno Pot, Colette Shortt, Wynand Alkema, Constance Bezulowsky, Stephanie Blanquet-Diot, Christophe Chassard, Sandrine P. Claus, Benjamin Hadida, Charlotte Hemmingsen, Cyrille Jeune, Björn Lindman, Garikai Midzi, Luca Mogna, Charlotta Movitz, Nail Nasir, Manfred Oberreither, Jos F. M. L. Seegers, Luc Sterkman, Audrey Valo, Frédérique Vieville, Magali Cordaillat-Simmons

**Affiliations:** ^1^Pharmabiotic Research Institute - PRI, Narbonne, France; ^2^MRM Health NV, Zwijnaarde, Belgium; ^3^Lallemand SAS, Blagnac, France; ^4^YUN Probiotherapy, Niel, Belgium; ^5^Accelsiors CRO, Budapest, Hungary; ^6^Department of Pharmacy, Faculty of Medicine, University of Novi Sad, Novi Sad, Serbia; ^7^Biose^®^, Arpajon-sur-Cère, France; ^8^Medical Affairs Department, ADM Protexin Ltd., Somerset, United Kingdom; ^9^Biofortis Mérieux NutriSciences, Saint Herblain, France; ^10^PharmaBiome AG, Zurich, Switzerland; ^11^Probiotical Research Srl, Novara, Italy; ^12^Science Department, Yakult Europe BV, Almere, Netherlands; ^13^Research Group of Industrial Microbiology and Food Biotechnology, Vrije Universiteit Brussel, Brussels, Belgium; ^14^Johnson & Johnson Consumer Services EAME Ltd., Foundation Park, Maidenhead, United Kingdom; ^15^NIZO Food Research B.V., Ede, Netherlands; ^16^IPSEN Pharma, Boulogne-Billancourt, France; ^17^University Clermont Auvergne, UMR 454 MEDIS UCA-INRA, Clermont-Ferrand, France; ^18^University Clermont Auvergne, INRAE, VetAgro Sup, UMRF, Aurillac, France; ^19^LNC Therapeutics, Bordeaux Cedex, France; ^20^Exeliom Biosciences, Dijon, France; ^21^Chr. Hansen A/S, Hørsholm, Denmark; ^22^Metabogen, Mölndal, Sweden; ^23^Astel Medica, Tinlot, Belgium; ^24^Lactosan, Kapfenberg, Austria; ^25^Caelus Health, Zegveld, Netherlands; ^26^Pilèje, Paris, France; ^27^5QBD-Biotech, Aydat, France

**Keywords:** toxicity, pharmabiotics, pharmacomicrobiomics, clinical development, safety

## Abstract

Recent developments in the understanding of the relationship between the microbiota and its host have provided evidence regarding the therapeutic potential of selected microorganisms to prevent or treat disease. According to Directive 2001/83/EC, in the European Union (EU), any product intended to prevent or treat disease is defined as a medicinal product and requires a marketing authorization by competent authorities prior to commercialization. Even if the pharmaceutical regulatory framework is harmonized at the EU level, obtaining marketing authorisations for medicinal products remains very challenging for Live Biotherapeutic Products (LBPs). Compared to other medicinal products currently on the market, safety assessment of LBPs represents a real challenge because of their specific characteristics and mode of action. Indeed, LBPs are not intended to reach the systemic circulation targeting distant organs, tissues, or receptors, but rather exert their effect through direct interactions with the complex native microbiota and/or the modulation of complex host-microbiota relation, indirectly leading to distant biological effects within the host. Hence, developers must rely on a thorough risk analysis, and pharmaceutical guidelines for other biological products should be taken into account in order to design relevant non-clinical and clinical development programmes. Here we aim at providing a roadmap for a risk analysis that takes into account the specificities of LBPs. We describe the different risks associated with these products and their interactions with the patient. Then, from that risk assessment, we propose solutions to design non-clinical programmes and First in Human (FIH) early clinical trials appropriate to assess LBP safety.

## Introduction

The development of molecular methods in recent decades has enabled the detection of non-cultivable microorganisms in different environments, including human and animal ecosystems, and has shifted the perception that most microorganisms are threatening, to a greater understanding of the importance of balanced microbial ecosystems in human and animal health. Consequently, new therapeutic approaches have emerged, aiming at re-establishing the necessary balance between the microbiome and its host in several pathologies. When such interventions are intended to prevent or treat diseases, in the European Union (EU) they fall under the definition of a medicinal product according to the Directive 2001/83/EC (1).

Medicinal products for which the active substance is a living microorganism, are currently being developed for multiple indications and are referred to by both the Food and Drug Administration (FDA) and the European Pharmacopeia (Ph. Eur.) as Live Biotherapeutic Products (LBPs) (2, 3). This type of product is defined as “*a biological product that (1) contains live organisms, such as bacteria; (2) is applicable to the prevention, treatment, or cure of a disease or condition of human beings; and (3) is not a vaccine*” by the FDA, and as “*medicinal products containing live micro-organisms (bacteria or yeasts) for human use*” by the Ph. Eur (which excludes fecal microbiota tranplants and gene therapy agents from this category). As for all biological medicinal products, LBPs represent a regulatory efficacy and safety challenge due to the live characteristics of the product and the often multifactorial mode of action (MOA).

The present review intends to provide an overview of the existing guidelines in the field of biological medicinal products, and to document how these guidelines can be used as a set of tools to assist in the design of an LBP development programme. At the same time we are proposing a road map that integrates crucial concepts laid down in existing documents directly or indirectly related to LBP-adapted safety assessment, in the absence of specific EU guidelines. Focus will be on how new techniques developed for discovery might allow more appropriate risk documentation and therefore better risk management in early clinical trials.

## Scientific Context

Complex microbial ecosystems inhabiting the human body are composed of bacteria, archaea, fungi, protozoa, and viruses, which altogether are known as “microbiota.” The term “microbiome” refers to the entire habitat of the microbiota, including the microorganisms, their genomes, and the surrounding environmental conditions (4). This galaxy of microorganisms within a multicellular host is the subject of intense interest for the biomedical scientific community (5). Large projects have focused on comparing the microbiomes of healthy subjects with the microbiomes of patients or at-risk populations. Demonstration of alterations in the microbiome composition (“dysbiosis”) supports the hypothesis that the microbiota is important in the maintenance of host homeostasis and that corrective intervention through LBPs may play a role in re-establishing the balance (6–8).

In order to design appropriate development programmes for this type of medicinal product it is important to understand how the microbiome is involved in the maintenance of human health and how LBPs may exert their beneficial effect. As mentioned, LBPs do not exert their biological effects by reaching distant organs, tissues or receptors, and, in most cases, do not act directly on a known target, but are thought to exert their effect by modulating the host microbiota, e.g., by inhibiting pathogens (9), producing active molecules/metabolites (9, 10), modulating the mucosal immune system activity (9, 11–13), activating cellular pathways within the epithelial cells (14, 15), or modulating the activity of the nervous system (16). Moreover, all or some of the above effects may occur simultaneously, which in turn, will mediate different types of signals, activating diverse physiological pathways within the host.

Importantly, LBPs will also exert their biological effect by influencing the local ecosystem, influencing other microorganisms and their interactions with the host, as conceptualized by the “holobiont concept” (17), or as explained by Foster et al. in a study on the common evolution between the microbiome and its host: “*unlike a rainforest or river ecosystem, the microbiome is not only driven from the bottom up by species interactions, but the host is under strong natural selection to shape the microbiota from the top down and foster a community that is beneficial*” (18). This co-evolution renders a full replication of all possible interactions extremely complicated, not in the least because they are host species-, even individual-specific (19).

Moreover, the environment (e.g., nutrition, stress factors, medications, etc…) is also an important parameter and has a large impact on the composition of the microbiota (19). Given this wider host-specificity, the translation of efficacy and safety signals from animals to humans is extremely difficult (20) since the likelihood for the conservation of LBP targets between species is very low. This is a concept of high importance when designing non-clinical programmes and in this respect, LBPs could present similarities with products currently classified as Advanced Therapies Medicinal Products (ATMPs).

## Importance of the Risk Analysis in LBP Development

The first mention of the LBP category at the European level was in the Ph. Eur. Monograph on LBPs published in 2019, which discusses the quality requirements for this type of products (3). In the EU no other specific guidelines exist to assist developers in their design of non-clinical and clinical studies for this type of medicinal product.

Fortunately, other European and international guidelines can be taken into account, like the International Council for Harmonisation of Technical Requirements for Pharmaceuticals for Human Use (ICH) Guideline on general considerations for Clinical Trials (ICHE8) (21), the Committee for Medicinal Product for Human use (CHMP) Guideline on strategy to identify and mitigate risks for first-in-human and early clinical trials with investigational medicinal products (22), and the CHMP guideline on Human Cell-Based Medicinal Products (23). These guidelines recognize that “*early clinical development of human medicinal products has an intrinsic element of uncertainty in relation to both the possible benefits and risks of a novel drug candidate. Uncertainty may arise from particular knowledge, or lack thereof, regarding the MOA, the presence or absence of biomarkers, the nature of the target, the relevance of available animal models and/or findings in non-clinical safety studies”* (22). These uncertainties will be reduced “*step-by-step by gathering relevant knowledge”* during the set of studies that sponsors and investigators will conduct on their medicinal products. Furthermore, competent authorities advise employing a “risk-based approach” to anticipate a priori “*the potential risk that might arise”* and to forecast “*appropriate risk mitigation strategies”* (22). Indeed, for cell-based medicinal products as well as LBPs, “*an initial risk analysis may be performed based on existing knowledge of the type of product, and its intended use. This should be updated by the applicant throughout the product life as data are collected to further characterise the risk. In addition, this comprehensive risk analysis should be used to justify the product development and serve as a basis for the preparation of the risk management plan*” (23).

**Figure 1:**
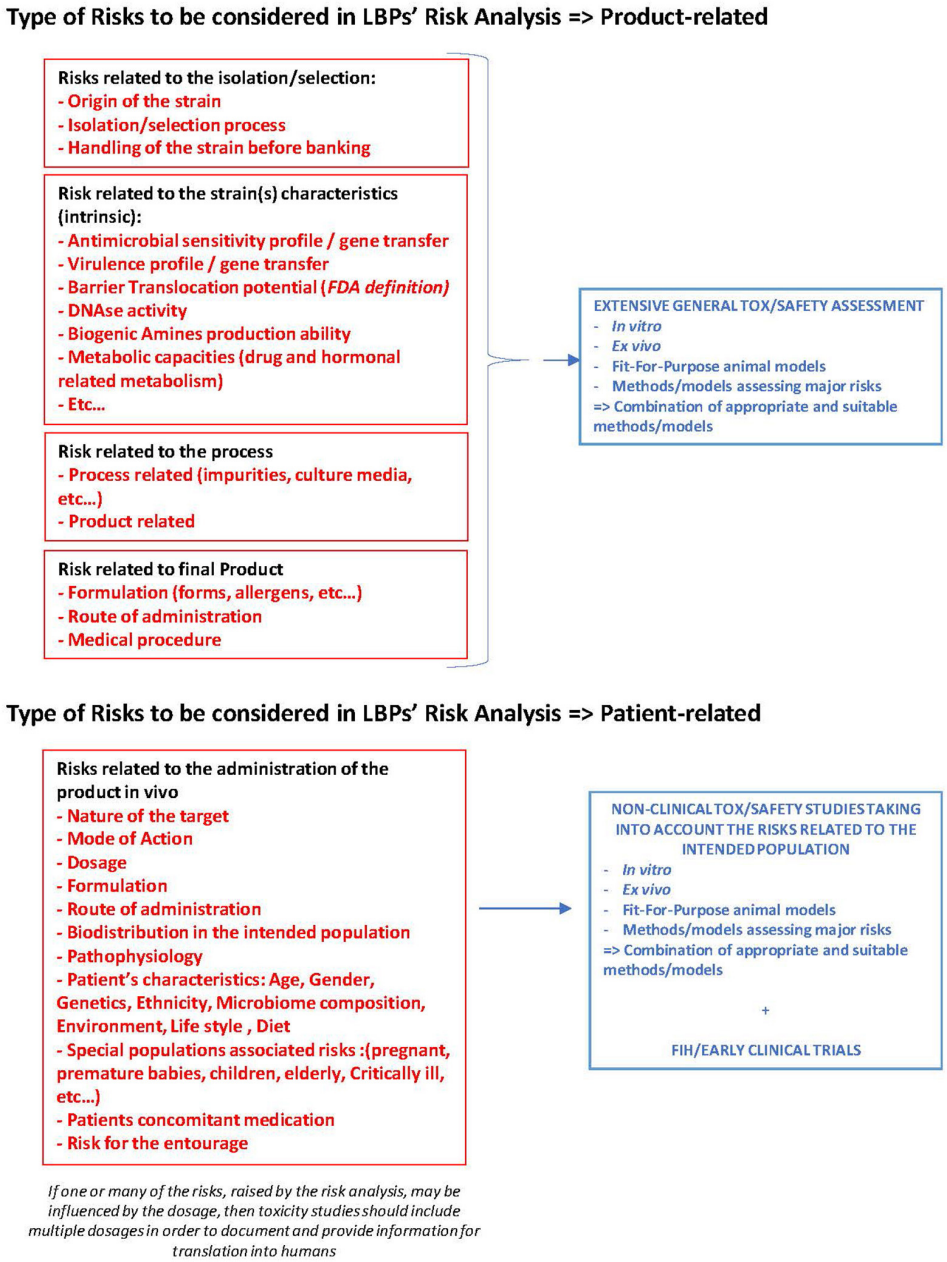
Road map for LBP's risk analysis. Different types of risks to be considered in LBP development and to be documented at the non-clinical and clinical stage.

For this purpose, risk analysis for LBPs should consider any risk intrinsic to the strain(s), as well as any information originating from the literature or the sponsor's data on the use of the strain(s) in different models or individuals (healthy humans or patients) and information on potential risks related to the particular characteristics of the intended population. The risks posed by the administration of LBPs may depend on the origin of the cells, the manufacturing process (e.g., culture media, microbial contaminants, impurities), the specific characteristics of the strain(s) (e.g., antimicrobial resistance, virulence, translocation ability, production of biogenic amines), and on the intended treatment population (e.g., influence of the environment, physiopathology, patient's microbiota composition). Figure 1 proposes an overview of the most important parameters to take into account when working on the risk analysis for LBPs.

Consequently, LBP developers should undertake a thorough risk analysis at a very early stage in development as it may guide the design of the non-clinical and clinical programmes, the outcomes to be monitored in the clinical trials, the definition of the risk management plan, and the contingency plan in case of severe adverse events in the intended population.

## Risk Documentation and Safety Assessment for LBPs

This section propose a risk analysis generally applicable to all LBPs, taking into account the microorganisms themselves and the characteristics of the intended population. Afterward, we will define and propose adapted tools for a non-clinical safety assessment of LBPs, allowing to subsequently improve the clinical programme designs.

### Characterization and Documentation of Risks Inherent to the Strain(s)

The potential safety issues associated with the administration of living microorganisms have been addressed in the past by different stakeholders, and identification and characterization at strain level has always been considered critical. However, research on microorganisms intended for food applications has shown that many safety-related aspects may be common at the species level (24). This principle has been endorsed by the European Food Safety Authority (EFSA), who uses a list, called the Qualified Presumption of Safety (QPS) list, expressing a species-based safety evaluation for microorganisms present in food (25, 26). It is important to note that the QPS approach was developed for products intended for healthy individuals and is therefore not appropriate for LBPs, which are intended to prevent or treat diseases. Where patients are concerned, a thorough risk/benefit analysis needs to be conducted, considering specific, relevant conditions and their management. Literature on the potential toxicity and pathogenicity of strains belonging to the same species as the product strain(s) can provide valuable supportive information for the design of safety studies and parameters to assess. However, documentation for a specific LBP should always be provided at the level of the strain(s) used as active substance.

Both the Ph. Eur. Monograph on LBPs (3) and the FDA guideline (2) highlight the importance of strain identification and characterization, since both therapeutic efficacy and safety profile of a drug product are active substance-specific (so in the case of LBPs strain(s)-specific). Strain characterization includes phenotypic and genotypic tests, documentation of the strain origin (and in the case of more recent isolates of human origin, e.g., based on the results and conclusions from large-scale comparative metagenomics, information pertaining to the health status of the original donor, if appropriate), and subsequent manipulation (passage history and generation of stocks), as outlined in the Ph. Eur. Monograph (3) and recommended by the FDA (27). Regarding strain characterization and documentation, the FDA currently expects whole-genome sequencing with *in silico* analysis for potential intrinsic risks (27). Both guidelines also stipulate that a description of the acceptance criteria and analytical methods, used to ensure identity, purity, and potency of the drug substance and drug product, are required as part of the LBP characterization (2). As stipulated by the FDA (27), strain characterization should focus on the identification of potentially undesirable traits of all microorganisms included in the product. In the EU and the USA, in order to set up post-market analysis, authorities also expect to be provided with information on the traceability of the strain(s) and donors.

A review of the important strain characteristics that should be considered when working on LPB's risk analysis is outlined below.

#### History of Use

In a therapeutic setting, the safety assessments of an LBP, based on history of safe use of its strain(s) in food, is not sufficient. Indeed, in Europe, post-market surveillance is not generally required for conventional foods and food supplements and adverse events in patient populations may not be systematically reported. In addition, in a 2011 report made by the US Agency for Healthcare Research and Quality (28), the authors concluded that although the existing clinical trials revealed no evidence of increased risk, the current literature cannot answer questions on safety in intervention studies with confidence. Wallace and MacKay (29) explained that, “*to address the question of safety using a drug-based framework, one assumes that drug-like safety and toxicology data are publicly available while this type of detailed safety data is often not included in clinical trial reports with foods*.” Indeed, in safety assessment of drug products, the physiology and pathophysiology of the target population must be taken into account. Consequently, while the history of safe use in healthy individuals may contribute to a demonstration of safety, documenting safety in the intended population requires a more profound assessment of population-specific parameters and risks.

#### Antimicrobial Resistance

Without any doubt, the antimicrobial sensitivity profile of the strain(s) present in the LBP is of the highest importance. There have to be sufficient options left for the patient to be treated with effective antibiotics in the event of an unexpected infection or allergy with the LBP. Combined approaches for the evaluation of the antimicrobial sensitivity profile should be considered taking into account the specificities of the intended population (including disease-specific characteristics as well as concomitant therapies) so as to optimize the assessment of the product's sensitivity to a relevant list of antimicrobials, considering the patient's pathology and relevant standards of care.

The EFSA regulatory framework therefore does not represents the right benchmark for sensitivity evaluation in patients.

For antibiotic sensitivity testing, it is recommended to complement molecular methods with culture-based methods. Also, a combination of different antimicrobial resistance databases should preferably be used in order to identify relevant antimicrobial resistance genes (22, 30): ResFinder (31), ARG-ANNOT (31, 32) and CARD (33, 34) or MARDy (35) for antifungal resistance genes. In addition, the Human Microbiome Project has also provided a large collection of antimicrobial resistance genes that can be used (36). For inherently present resistances, the absence of transferability should also be demonstrated convincingly (2).

In relation to the culture-based antimicrobial sensitivity profiles, the FDA regulatory guideline for LBPs (2) requires the determination of the Minimum Inhibitory Concentrations (MIC) values or the Minimum Bactericidal Concentration (MBC). If no MIC value can be defined, the therapeutic value of the antimicrobial should be assessed. No specific method is mentioned in this guideline, but standard methods for the antimicrobial susceptibility testing of clinical isolates [e.g. the ones from the Clinical and Laboratory Standards Institute (CLSI)] (37, 38), have been recognized by the FDA. Harmonization of the interpretation of MIC values for LBPs is needed at the EU level as the European Committee on Antimicrobial Susceptibility Testing (EUCAST) only monitors MIC values for clinical isolates. Clearly, their involvement in the compilation of a dedicated guideline and list with reference MIC values for LBPs would be very beneficial, offering dedicated pharmaceutical guidelines and harmonized cut-off values to companies and authorities.

The WHO has developed and applied criteria to rank 35 classes of antimicrobials into three categories according to their relative importance in human medicine. In particular, the sixth revision ranks antimicrobials as “Critically Important,” “Highly Important,” and “Important.” Critically Important antimicrobials are the ones which are either the sole, or one of the limited, therapies to treat serious bacterial infections in people, or are used to treat infections caused by bacteria possibly transmitted from non-human sources, or with resistance genes from non-human sources. While the list mainly concerns the non-human use of antimicrobials, it could be used as a useful reference to establish a list of relevant antimicrobials for LBP safety assessment, considering the current standard of care and precautions in the intended population. Moreover, the list could also become the basis for genomic predictive scouting, whereby positive hits could lead to further *in silico* and *in vitro* MIC determination (39, 40).

When resistance is found to be acquired or intentionally introduced, rather than intrinsic, the extent of the risk of transmission to other microorganisms of the patients' microbiota, as well as the measures taken to mitigate this risk, should be documented as such potential transfer may create a long-term safety concern for the individual and the wider public. With respect to the assessment of potential transfer risk, harmonization and international standards would also be very valuable to the industry to ensure consistency in product development, and would facilitate the evaluation of dossiers by the competent authorities.

#### Virulence Factors

Virulence is the potential of a microorganism to harm its host. The pathogenicity of an organism is generally determined by its virulence factors, including proteins or molecules produced by the microorganism, allowing it to evade the immune system, to colonize the host, or to produce toxins. Typically, these factors can be neutral, offensive (e.g., flagella, toxins), or defensive (e.g., acid resistance, antibiotic resistances, etc.). Microorganisms acquire these factors often through vertical or horizontal gene transfer.

The degree of virulence of a particular organism may depend on the host's physiology and immune status. Known virulence genes can be found through *in silico* searches of the annotated genome sequence using relevant databases (41–43). The FDA guideline on LBPs (2) considers the assessment of virulence genes to be part of the characterization of the microorganism and requires developers to provide methods to attenuate a virulent strain, as well as document the stability of such attenuation (2). Similarly, in the EU, the Ph. Eur. Monograph on LBPs states that the presence of virulence factors must be investigated and evaluated with respect to safety (3). If virulence factors are identified, their potential risk of transfer to the microbiota should be assessed as this may represent an important safety concern for the patient.

Since the present knowledge on virulence genes in yeasts is not as extensively developed as for bacteria, Anoop et al. (44) suggested a polyphasic approach combining genetic, *in vitro* and *in vivo* exposure studies to identify the pathogenicity of industrial and biotherapeutic strains of, e.g., *S*. *cerevisiae*.

Overall, excluding known human pathogens, the chances of finding putative virulence factors through genome scouting is highly likely for most bacterial isolates. Therefore, interpretation of the results needs to be further contextualized within the framework of the strain's phylogeny and the health condition of the intended patient. The latter is critical, as the vulnerability of the host is likely more important than the presence of specific virulence traits (45). Once more, recommendations in this area could be very valuable, allowing to harmonize the requirements of virulence testing of LBPs developed for many different indications, including pathologies where patients are immuno-compromised.

#### Translocation

One of the most important risks associated with the administration of living microorganisms is translocation. Bacterial translocation in the gut is defined as the passage of members of the gastrointestinal microbiota across the *lamina propria*, to the local mesenteric lymph nodes and beyond (46). It has been suggested as a direct cause of infection and inflammation, which, in certain conditions, may predispose to the development of sepsis and subsequent organ failure (46).

In healthy individuals, controlled, physiological translocation may be a desirable phenomenon, without deleterious consequences (47, 48), allowing the gut to be exposed to antigens and to develop a certain level of tolerance (48), e.g., against the native microbiota, or to prepare for immunological action against detected pathogens. However, uncontrolled translocation, especially when associated with bacterial overgrowth, barrier damage or immunosuppression, can have severe consequences in patients (48). For this reason, the ability of the product strain(s) to cross the mucosal barrier becomes a critical safety concern. With respect to the relationship between translocation potential and pathogenicity, two aspects should be addressed: (1) the ability to cross a mucosal barrier, and (2) the potential to induce a pathogenic reaction upon passage to the systemic circulation (inflammation- or bacteria-mediated organ damage). Indeed, the clinical relevance of bacterial translocation in the pathogenesis of sepsis and organ failure is still controversial, as they can occur independently of each other (49).

Therefore, documentation of translocation potential remains challenging, as it requires the integration of multiple parameters relating to the host as well as to the strain(s). FDA considers the assessment of the translocation potential as part of the characterization of the strain and recommends the use of a reproducible translocation assay, preferably in an appropriate animal model, such as germ-free mice (2). Assessment, however, remains difficult because of the lack of conservation of immune and mucosal targets between species (animal vs. human), leading to very laborious experimentation in these animal models with no certainty of the relevance of the outcome in the human situation.

Examples of more global approaches for this challenging assessment can be found in the literature which will help LBP developers in their efforts to document their strain(s). In regards to the assays developed for the evaluation of both abilities mentioned above (that of crossing a mucosal barrier and inducing a pathogenic reaction), examples are provided by Holzapfel et al. on *Enterococcus faecium* SF68, a strain contained in an LBP registered nationally in Switzerland since 1979 (50). SF68 was found to maintain physiological epithelial cell structure in *in vitro* experiments performed on porcine jejunal epithelial cell lines (IPEC-J2) that were challenged with enterotoxigenic *Escherichia coli* (ETEC). Additionally, when immuno-compromised mice received persistent exposure to large intravenous doses of SF68 no traces could be detected in liver, kidney or heart after plating individual homogenized organs.

Daniel et al. also used marked strains from food or infection sources and administered these to healthy mice without any consequence. When administered to animals with a damaged mucosal barrier, induced by the administration of TNBS (2,4,6 trinitrobenzene sulfonic acid), only strains from infection sources were found to translocate to the different organs in the mice, while food-derived strains did not (51). This experiment illustrates the strain specificity of the translocation potential as well as the importance of the host's status and barrier integrity. Clearly both elements need to be considered in the LBP development pipeline. In this regard, the influence of the LBP on the mucosal barrier could provide valuable information, as mucosal barrier disruption is known to be a risk factor, while a positive influence on the mucosal barrier integrity could be considered an argument in favor of the safety of the product. Animal studies, especially those with an induced damage to, e.g., the mucosal barrier, might, for ethical reasons, no longer be considered appropriate models. Still the safety in diseased or at-risk populations needs to be investigated according to the existing regulation. The development of 3D cell culture techniques or organ-on-a-chip developments may in the future replace these animal models.

#### Particular Metabolic Activities and Potential Drug-Drug Interactions

The full understanding of the MOA of a living microorganism used as an LBP is not (yet) a requirement for its registration as a medicinal product if quality, safety and efficacy have been demonstrated and documented through appropriate clinical trials. However, bearing in mind the recent trend to also apply drug quality standards to the production of biological medicinal products, knowledge or partial understanding, of the MOA may become a prerequisite (52). Indeed, in the context of a “Quality by Design” (QBD) approach, Critical Quality Attributes (CQAs) monitored along the manufacturing process are defined in relation to the safety and efficacy of the product. These CQAs are physicochemical or biological characteristics, often based on the partial understanding of the MOA, as well as the intrinsic characteristics of the strain(s), and/or particular characteristics of the patient. In this context, research on the MOA is of value as it allows the appropriate definition of CQAs and the corresponding assays, including assays relating to safety.

It is also commonly accepted that living microorganisms may exert their biological effect (positive or negative) through several, potentially simultaneous, direct or indirect MOAs which may depend on the specific host environment. While this complexity represents a challenge for LBP developers, understanding the potential capabilities of the strain(s) and their MOA remains highly valuable as it may further help to document the risks and benefits by identifying the potential negative or positive secondary pharmacodynamic effects (MOA and/or effects of a substance not related to its desired therapeutic target).

To illustrate the importance of understanding the MOA in terms of safety, we can mention the case of biogenic amines (BAs) and drug-metabolizing enzymes, which are intrinsic characteristics of the strains, and should be documented as a risk pertaining to potential drug-drug interactions.

##### Drug metabolizing enzymes

Human commensal bacteria are now known to be capable of metabolizing drugs and/or drug metabolites affecting the pharmacokinetics of the drug. This phenomenon, known as “pharmacomicrobiomics,” is considered to play a major role in the efficacy and toxicity assessment of drugs (53–58). Zimmermann et al. evaluated the capacity of 76 human gut bacteria to metabolize 271 oral drugs and mapped the genetic footprint of drug metabolizing enzymes in these bacteria (59). Future safety screening tests of LBP candidates need to address and document the potential presence of drug metabolizing enzymes, considering that these products will likely be consumed concomitantly with a range of pharmaceuticals that are affected by such enzymes. This will be especially important *in situations* of polypharmacy, such as in severe acute diseases (e.g., sepsis), complex chronic diseases (e.g., cancer), and in elderly individuals. In order to improve the value of a specific dossier, an approach similar to a “drug-drug interaction” investigation could be used to test LBP impact on relevant drugs or known biological markers of a specific disease. In cases where such potential is expected, monitoring of the kinetics of the drug during the first human trials would allow for the definition of appropriate risk management measures.

##### Biogenic amines

BAs, such as histamine and tyramine, are low molecular weight organic molecules with one or more amine groups, commonly detected in many foods and beverages. Exogenous BAs can be formed by enzymes in plants and animals, but can also be generated in significant concentrations by certain microorganisms through the activity of amino acid decarboxylases (60). Endogenous amines are produced by the host itself, e.g., histamine by mast cells or liver cells.

While strains from different Gram negative and Gram positive genera harbor the capacity to decarboxylate amino acids and produce BAs, lactic acid bacteria are the main producers (61). BAs play an important role in cellular physiology; therefore, their concentration is carefully regulated. High intake of BAs can induce several digestive, circulatory and respiratory symptoms, and the severity of which depends on the amount, the variety ingested, the individual susceptibility and the level of detoxification activity in the gut (61). The enzymes Mono-Amine Oxidase (MAO) and Di-Amine Oxidase (DAO) effectively detoxify BAs but the level of detoxification might be influenced by MAO inhibitors and DAO inhibitors, leading to more severe toxicity in patients treated with such a type of antidepressant (61). In the case of LBPs, metabolic pathways potentially leading to BA formation should therefore be assessed, taking the patients' population characteristics into consideration. Particular attention should be given to the patients' sensitivity to BAs, including their drug use.

### Non-clinical Documentation of the Risks Emerging From the Administration of LBPs

As mentioned in the ICHS6(R1) guideline, “*conventional approaches to toxicity testing of pharmaceuticals may not be appropriate for biopharmaceuticals due to the unique and diverse structural and biological properties of the latter that may include species specificity, and unpredicted pleiotropic activities*.” In the microbiota field as well, developers have to be innovative in their approach for risk documentation, and therefore safety and toxicity assessment. The main challenge is the limited relevance and poor predictability of *in vivo* animal models (20, 62, 63), as discussed before. Since the microbiome-host symbiosis is highly complex and highly species-dependent (18, 19) any difference in the microbiota composition may have substantial consequences on host physiology (63). This renders the translation of efficacy and safety signals from animals to human extremely difficult, as is also the case for most biotherapeutics.

We therefore believe that it is important for developers and competent authorities to address LBP safety through a combined approach. As highlighted in the work of the EU reference Laboratory for alternatives to animal testing (EURL ECVAM) regarding alternative methods on toxicity testing for chemicals (64), integrated approaches to toxicity assessment are based on the integration and translation of data derived from multiple methods and sources (65). The same spirit of the initiative could also be applied to the development of LBPs. For LBPs with topical administration, alternative validated methods provided by ECVAM (66–68) could be considered in the safety assessment.

Above all, the usefulness of any model for toxicity and safety testing, should be evaluated in terms of their suitability in line with the MOA and the associated risks, their reproducibility, as well as their predictability in terms of safety in the intended population. If mammalian models are used as part of an integrative safety documentation approach, their selection should be based on a deep understanding of the relationship between the targeted host and his/her microbiota. This includes knowledge on the pathophysiology of the targeted (patient) population, the MOA of the LBP, and knowledge about the degree of similarity of the microbiota between the animal model and the intended host in terms of composition and function, as well as the potential conservation of the LBP target.

#### Newly Developed Research Tools for LBP Risk Documentation

##### In vitro tools

According to ICHS6(R1), “*biological activity may be evaluated using in vitro assays to determine which effect of the product may be related to clinical activity*.” The guideline also refers to the “*examination of direct effects of the product on cellular phenotype and proliferation through the use of cell lines and/or primary cell cultures*” (69). In the case of LBPs, similar models could provide valuable information on the MOA of the product when a direct effect of the microorganism(s) on the host cell is to be expected, but cannot be used when the biological effect(s) of the LBP is(are) indirect, e.g., a change of the ecosystem composition impacting host physiology. In order to deal with this complexity, new assays, involving microorganisms and human intestinal cells are currently being developed. The pros and cons of the different technologies, as reviewed by Pearce et al. (70) or Kang et al. (71), should be taken into account when addressing the usefulness of these new models in the context of safety assessment in relation to the intended target population and the product MOA. Techniques like organs-on-chip and microfluidic devices containing human cells (73), could accurately reproduce the human physiology and the interactions between human cells and bacterial communities.

Similar *ex-vivo* models have been recently reviewed for products intended for topical applications (74). 3D-skin models or skin-on-chip technologies, coupled to microfluidic culture devices, may provide new methods to assess the effect of LBPs on human skin. Models reproducing healthy, wounded, or diseased skin have been used to assess the impact of commensals and pathogenic strain(s) on keratinocytes as well as on inflammatory responses to bacteria (74).

As for other routes of administration, recent developments in the field of the vaginal microbiota have also led to new models and technologies, including monolayers or 3D models, as well as aggregates displaying *in vivo*-like features (75).

Finally, an important drawback for all of these innovative models is that they have not yet been validated for use in drug development, while there is even less data available for LBPs. However, these models could be considered as potential options for developers as the FDA will consider them favorably, as highlighted in the recently released information sheet (76).

##### In vivo tools

*Simple animal models*. When looking at *in vivo* models for LBP risk documentation, simple animal models like *Drosophila melanogaster* (fruit fly) or *Caenorhabditis elegans* (nematode worm) might be helpful in, e.g., deciphering pathways within the host. As reviewed by Gerbara et al. (77), *C. elegans* could allow for the construction of defined consortia targeting specific biological processes or for deciphering conserved molecular pathways relevant to mammals. It could also be a suitable model to understand whether the host, the microbe, or the environment, determines the susceptibility or resistance to infections. Therefore, the model could provide insights into mechanisms that underly potential adverse reactions of LBPs when interacting with the host and/or native microbiota. Immunodeficient *D. melanogaster* could also be used to better understand mechanisms involved in the possible transition from symbiotic to pathogenic microorganisms (78, 79) and could also offer a platform to screen *in vivo* microbe-xenobiotic interactions, providing insight into safety outcomes potentially related to the metabolism of regular drugs by the microbiome (79). Even if it remains difficult to translate these results to humans, these models do provide promising and ethically more acceptable alternatives than mammalian models for the preliminary safety testing (or screening) of candidate LBPs (80).

*Mammalian animal models*. Due to the limited relevance and poor predictability of *in vivo* animal models for the human situation (20, 62, 63), the use of humanized animals has also been discussed (81). While some improvement of the animal to human translation factor can be expected, the facts that (1) the host did not co-evolve with the introduced microbiota, and (2) the animal species may never have been in contact with the human microbiota or the LBP's strain(s), responses might still not be completely representative of the complex interactions. In addition, the human microbiota transplanted to animals may evolve toward a composition that is closer to what is normally observed in the recipient host, albeit not fully returning to the natural composition and, therefore creating confounding factors (81). Other influencing factors, such as the feed provided to the animals, the way they are handled in the facilities (e.g., hygiene conditions or cage differences), or even the origin of the animal (vendors) may at least in part be responsible for what happens to the microbiota composition after transplantation and the subsequent experimental results (82). Finally, Walter et al. warned that the very high success rate of phenotype transfer from pathological humans to recipient animals (95% of the published studies) is quite unlikely and might overstate the impact of the gut microbiome in human disease, rendering the potential of these humanized animal models questionable as well (83).

When designing non-clinical programmes, these aspects should be taken into account, as well as the 3R ethics rule (Replacement, Reduction and Refinement) (80) which strongly encourages the development of alternative methods in order to decrease the number of animals used in research. This is also in line with the CHMP Guideline on strategy to mitigate the risk for FIH studies and early clinical trials with investigational medical products (22) which states that “*for biotechnology-derived products, and in line with ICH S6(R1), studies in non-relevant species may give rise to misinterpretation and are discouraged*,” and the CHMP guideline on human cell-based medicinal product, mentioning “*if relevant animal models cannot be developed, in vitro studies may replace animal studies*” (23).

##### Dose selection for LBPs

An important aspect of non-clinical studies is the assessment of the potential risks associated with the dosage regimen. These programmes should be designed in order to provide information on the dose to be used in clinical trials, the route of administration, the administration schedule, the duration of exposure and the duration of the follow up period during which adverse reactions are to be tracked.

Biodistribution is key information when addressing the question of the dosage for LBPs and the potential risk estimation associated with the behavior of the strain once administered. Studies should monitor the effective presence of the strain(s) at the site of action, assess potential engraftment (at the site of action or in other locations), as well as the elimination after administration has ended.

LBPs are not expected to reach the systemic circulation, therefore, conventional Absorption, Distribution and Metabolism (ADM) studies are not relevant for these products. Once again, LBPs share similarities with human cell-based medicinal products, for which the guideline (23) does not require conventional pharmacokinetic studies, but specifies that “*studies should be carried out to demonstrate distribution, viability, trafficking, growth, phenotype and any alteration of phenotype due to factors in the new environment*.”

When efficacy is mediated by indirect mechanisms of action, e.g., by impacting unidentified microbiota components at the site of action, the dosage of the product might be partly disconnected from the level of efficacy and safety, and may depend on the individual's physiology (stomach pH, digestive enzyme production, intestinal bile concentrations, etc.) and ecosystem composition and complexity. The assessment should therefore include models taking into account the patient's characteristics and his/her physiology. Gut indications are the most challenging in this context as animal models may be poor predictors of the actual behavior of LBP's in the human Gastrointestinal Tract (GIT), and because the monitoring of their presence at the site of action requires rather invasive methods. Artificial models of the human GIT have, however, been optimized and validated for microbiota research and represent an interesting alternative. Some examples of currently available systems are the SHIME^®^ (ProDigest and Ghent University, Gent, Belgium) and the TIM^®^ (Triskelion, Zeist, The Netherlands and Clermont Auvergne University, Clermont Ferrand, France) models.

Both systems represent a relevant alternative to animal models and allow the assessment of survival kinetics and distribution of strain(s) (beneficial or pathogenic) in the GIT under different (patient-like) conditions and with different formulations, as well as the evaluation of their influence on the human microbiota as published (84, 85) for the TIM^®^ and (86) for the SHIME^®^ system. Importantly, both models allow for the inoculation with fecal samples of patients and healthy controls, representing a valuable alternative to human sampling through endoscopy or other invasive procedures. Both systems have been used for assessing the survival and virulence of pathogenic strain(s) under different conditions (84), as well as to investigate the possible influence of an LBP (87). In cases where assessing the presence of a specific strain (or strains) in feces through PCR does not indicate the actual viability of the product, these models may provide information on viability, metabolic activity, potential engraftment and growth of the product along its passage through the GIT. The models may also provide valuable information on MOA or potential unfavorable behavior of an LBP, such as conditions that mimic the patient's unique gut environment, and may provide relevant information on the efficacy and safety of an LBP in relation to different dosages, formulations, durations of exposure, or durations of the follow-up period, and may yield safety outcomes under conditions that mimic the patient's gut condition.

This information could help developers to narrow down their Optimal Effective Dose Range (OEDR), defined as the largest dose range required to obtain the intended effect based on the clinical results for efficacy and tolerability, before Phase I/II clinical trials.

If animals are chosen for biodistribution studies, their suitability should be accompanied by the documented rationale that supports the translation and extrapolation of the dosage from animals to humans.

In conclusion, non-clinical studies are inherently part of an LBP development cycle where, in comparison to traditional pharmacodynamic and pharmacokinetics studies, many adaptations will have to be made. Harmonized guidelines at this level may be useful in guiding developers in their risk analysis and in the design of non-clinical studies which will then provide a picture as complete as possible of the safety profile of their LBPs.

To discuss the acceptability of these innovative models, developers should seek scientific advice from competent authorities.

### Safety Considerations in Early Clinical Trials With LBPs

It can be expected that the information on LBPs' safety will often be provided mainly by early clinical trials as they are the only way to test the products in the target environment where the complex interactions between the host and his-her co-evolved microbiota are present.

The main objective of the early clinical phases is to define the appropriate dosage range and the administration schedule to be used in confirmatory clinical trials based on the tolerability of the product. It is important to remember that the various risks associated with LBPs may not always be directly related to the dosage as they depend highly on the host-microbiota interactions, the patient's mucosal barrier integrity and the host's immune status. As cell-based therapies are in a similar situation, we can therefore apply similar concepts in the design of the early clinical trials for LBPs: “*Phase I/II studies should be designed to identify the Minimal Effective Dose, the lowest dose to obtain the intended effect, or an Optimal Effective Dose Range. If possible, also a Safe Maximal Dose, defined as the maximal dose which could be administered on the basis of clinical safety studies without acceptable adverse effect, should be investigated*” (23). Moreover, the definition of the Safe Maximal Dose (SMD) should also take into account the possibility of a repeated administration (23) as most of these products are intended for the treatment of chronic diseases.

Another point regarding study design adaptation is the potential bias in safety assessment during early clinical trials with healthy volunteers. For strain(s) isolated from healthy humans, it can be expected that some healthy volunteers might carry, if not the same, at least some representative strain(s) from the same species in their native ecosystem, or carry strains that provide functional redundancy (88, 89). Consequently, phase I studies that enroll patients rather than heathy volunteers are, in our opinion, more appropriate, especially when LBPs have been developed to, e.g., correct a large dysbiosis affecting certain species in the patient population. If the LBP strain(s) has no history of use in humans, and if no other risk has been identified during non-clinical development, it is therefore upon the developer to put in place appropriate risk mitigation measures in the target population, such as sequential enrolment, dose escalation, setup of an independent Data Monitoring Committee, etc…

For LBPs, it is also important to take into account the influence of other confounding factors, such as environment, diet, medications and (other) nutraceuticals, food products containing living microorganisms or prebiotics, genetic background, and geographical location, all of which could have an influence on the composition and/or function of the microbiome. Tolerability studies with placebo-controlled cross-over designs should be considered as an option to exclude the influence of such extrinsic and intrinsic factors, as patients become their own controls. In such designs, however, blinding will be important and the “wash-out” period will have to be carefully considered. Moreover, interventions designed to, e.g., (permanently) correct a dysbiosis cannot use this type of cross-over set-up, as a return to the base line is by definition no longer possible.

For long-term use of LBPs, patients consenting may benefit from the biobanking of their samples obtained during the different clinical phases, as they could be valuable for future assessments of long-term effects that might not have been anticipated at the time of trial. These samples might therefore allow to further optimize individual treatment strategies, to design future studies directed toward obtaining a better understanding of the mechanisms involved, as required under the pharmacovigilance rules, or assist in clarifying subgroups of, e.g., responders and non-responders.

Finally, as specified in the CHMP guideline on strategies to identify and mitigate risks for FIH studies and early clinical trials with investigational medicinal products (22), risk mitigation procedures and stopping rules should be defined, taking into account the body of knowledge acquired from non-clinical programmes, literature, patients' characteristics, and normal standard of care of patients. The risk analysis and knowledge of the LBP and of the patients' characteristics are key to addressing each situation. Immunocompromised populations (Young, Old, Pregnant, Immune deficient; YOPIs) are obviously of concern and, as for all special populations, the strategies in place to mitigate and manage the risk, and the accompanying contingency plan are key in the design of clinical trials for these patients. Analysis of the specific guidelines regarding different special populations is encouraged in the light of the particular risks identified for the LBP. However, the nature of LBPs does not have any influence on the rules to be applied when designing clinical trials with these populations (90–94).

Potential metabolic activities of the strain(s) are important parameters to consider in the case of strain(s) presenting a strong potential for the production of BAs, bloating, diarrhea and stomach pain should be commonly monitored for LBPs with gut indications.

On the other hand, the potential metabolism of non-related drugs by the microbiota is also becoming a topic of concern (59, 95) as there is growing evidence that many drugs, such as antacids, prokinetics, antispasmodics, antibiotics, laxatives, antipsychotics and antineoplastic agents, can affect the microbiota (56). This should not be a topic of concern at the stage of the FIH studies which in theory are carried out on healthy volunteers, however, as explained above, as it seems more appropriate to test LBP tolerability and safety in the target patients, it might be unethical to withdraw them from their medication. Consequently, in these particular situations (Phase I or II), the kinetics of the respective maintenance drugs should be assessed as part of a drug-LBP interaction assessment.

Similarly, the microbiota may impact the metabolism of different hormones (72) thereby potentially affecting some of the associated physiological functions. If LBP influences on host hormonal physiology are likely, they may represent a risk that requires tolerability studies to assess the hormonal status of the patients before, during and after treatment.

Nevertheless, the main risk identified for all LBPs, regardless of indication or site of action, is the potential for translocation, migration, and infection of distant organs as discussed above. This risk highly depends on the host and the monitoring is of paramount importance in instances of poor barrier integrity, or if the patients present an impaired gut motility (and/or e.g., bacterial overgrowth) or are immunocompromised (YOPI). Clinical outcomes relating to such risks, like routine body temperature recording, could allow for early detection and early mitigation, including an immediate stop of the administration and/or the treatment with an appropriate antimicrobial for which the LBP had been proven sensitive during the non-clinical characterization phase of the development.

Overall, the body of knowledge acquired during non-clinical programmes, pertaining to potential risks associated with the strain(s), the product, or the intended population, should guide developers while designing early clinical trials and the appropriate management and contingency plans.

## Conclusion and Actionable Recommendations

In the absence of any EU guideline on the development of LBPs, developers are strongly advised to discuss the development process with regulatory authorities, especially as this field is rather new. In the EU this can be performed via the so-called Scientific Advice Procedure. This should include proposals for the most suitable studies guaranteeing sufficient quality and (pre)clinical safety, and it should help developing protocols to demonstrate efficacy. These products represent a real challenge, because their MOA does not rely on their absorption in the systemic circulation nor on a simple direct liaison between a ligand and a receptor, but is rather related to an impact on the local ecosystem and the local host cells, involving various direct and indirect mechanisms. Thus, existing CHMP and ICH guidelines (22, 23, 52, 69) developed for other types of biologicals, can provide important information when designing non-clinical and clinical developments for LBPs. The present discussion aimed at providing some insights on how developers can envision safety development programmes for their products, considering the specific nature of LBPs and the often particularly complex nature of their MOA. Efforts were also made to provide examples from the literature of alternative methods and models which could be more appropriate for the documentation and development of this type of medicinal product.

As new products are being developed, we hope that this review will improve the understanding by all stakeholders, including government and regulatory agencies, and advance the regulatory harmonization, resulting in the development of dedicated and specific guidelines and recommendations for LBP-specific drug and microbiome-based medicinal product development programmes. This will increase consistency in LBP development and encourage investment/funding for developers, offering new and promising microbiome-based therapeutics for various diseases and many patients in the future.

## Author Contributions

AR contributed to the drafting of the manuscript as first author. SB, AB, IC, HC, HG, AH, SL, SM, MP, BP, and CS equally contributed to the discussions and the drafting of the manuscript and are equally considered as second authors of the manuscript. WA, CB, SB-D, CC, SC, BH, CH, CJ, BL, GM, LM, CM, NN, MO, JS, LS, AV, and FV equally contributed to the discussions and provided some input along the drafting of the manuscript. MC-S contributed to the discussions and to the drafting of the manuscript as last author and corresponding author.

## Conflict of Interest

The opinions expressed herein, and the conclusions of this publication are those of the PRI and authors alone and do not necessarily represent the views of their member companies. SB is employed by the company MRM Health NV. AB is employed by the company Lallemand SAS. IC is employed by the company YUN Probiotherapy. HC is employed by the company Accelsiors CRO. HG and CJ are employed by the company Biose. AH and GM are employed by the company ADM Protexin. SL is employed by the company Biofortis Mérieux NutriScience. SM is employed by the company PharmaBiome AG. BP is employed by the company Yakult Europe BV Science Department. CS is employed by the company Johnson & Johnson Consumer Services EAME Ltd. WA was employed by the company NIZO Food Research B.V. CB is employed by the company IPSEN Pharma. SC is employed by the company LNC Therapeutics. BH is employed by the company Exeliom Biosciences. CH is employed by the company Chr. Hansen A/S. BL and CM are employed by the company Metabogen. MP and LM are employed by the company Probiotical Research. NN is employed by the company Astel Medica. MO is employed by the company Lactosan. JS and LS are employed by the company Caelus Health. AV is employed by the company Pilèje. FV is employed by the company 5QBD-Biotech. The remaining authors declare that the research was conducted in the absence of any commercial or financial relationships that could be construed as a potential conflict of interest.
